# Aortic valve morphology and paravalvular leak regression after a self-expandable transcatheter aortic valve replacement

**DOI:** 10.3389/fphys.2022.1088681

**Published:** 2023-01-06

**Authors:** Qinchun Jin, Wei Li, Shasha Chen, Mingfei Li, Daxin Zhou, Xiaochun Zhang, Junbo Ge

**Affiliations:** ^1^ Department of Cardiology, Zhongshan Hospital, Shanghai Institute of Cardiovascular Diseases, Fudan University, Shanghai, China; ^2^ National Clinical Research Center for Interventional Medicine, Shanghai, China; ^3^ Department of Echocardiology, Zhongshan Hospital, Fudan University, Shanghai, China

**Keywords:** transcatheter aortic valve replacement, aortic valve morphology, paravalvular leak (PVL), bicuspid, tricuspid, calcification

## Abstract

**Aims:** The study aimed to compare paravalvular leak (PVL) changes after a transcatheter aortic valve replacement (TAVR) with self-expandable prosthesis between different aortic valve morphologies and evaluate the impact of paravalvular leak regression on clinical prognosis.

**Methods:** Patients with aortic stenosis (AS) successfully treated with a self-expandable TAVR who were followed up for at least 1 year at our centre were consecutively enrolled from January 2016 to August 2019. Paired serial changes in paravalvular leak and other haemodynamic parameters by echocardiography were collected and compared between the bicuspid valve (BAV) and tricuspid aortic valve (TAV). A logistic regression model was used to explore the predictors of paravalvular leak regression (<1 grade) 1 year after transcatheter aortic valve replacement, while its impact on subsequent clinical outcomes (all-cause mortality and rehospitalisation for heart failure (HF)) was further evaluated using Kaplan–Meier analysis.

**Results:** A total of 153 bicuspid valve and 114 tricuspid aortic valve patients were finally enrolled; haemodynamic parameters and paravalvular leak severity were comparable before the discharge between the two groups. The peak transaortic velocity, mean transvalvular gradient, and effective orifice area all significantly improved (*p* < 0.05) without intergroup differences at all follow-up timepoints. Significant paravalvular leak reduction was observed only in the TAV group (1.75% *vs.* 4.39%, *p* = 0.029), while moderate paravalular leak was still more prevalent in BAV (7.19% *vs.* 1.75%, *p* = 0.041) at the 1-year follow-up. Multivariable analyses identified the bicuspid valve, asymmetric calcification, and undersizing as independent predictors of failure of the 1-year paravalvular leak reduction in patients with mild or moderate paravalvular leak after discharge. Patients without a paravalvular leak reduction within 1 year showed a relatively higher 2-year all-cause mortality and HF (HR: 5.994, 95% CI: 1.691–21.240, and *p* = 0.053) rates thereafter.

**Conclusion:** In AS patients after self-expandable transcatheter aortic valve replacement, paravalvular leak regression within 1 year was less prevalent in bicuspid valve morphology. The failure of paravalvular leak reduction might lead to an increased risk of poorer prognosis in the long run.

## Introduction

Over the past decades, advances in transcatheter aortic valve replacement (TAVR) have led to its consideration as an optimal alternative to surgical treatment for severe aortic stenosis (AS). To date, researchers have demonstrated that self-expandable valves and balloon-expandable valves have a comparable clinical efficacy and safety (Abdel-Wahab et al., 2014; Lanz et al., 2019; Deharo et al., 2020), and additional improvements in aortic valve haemodynamics in SEV have been observed over time (Kaya et al., 2016; Shishido et al., 2019).

According to Oh et al. (2015), the CoreValve U.S. Pivotal Trial demonstrated the continuous improvement in echocardiographic parameters and regression of residual aortic regurgitation, following CoreValve implantation in TAV patients. Their findings were consistent with studies on valve performance, following TAVR using other self-expandable prostheses in the TAV upon longitudinal echocardiographic follow-ups (Sondergaard et al., 2018; Popma et al., 2019; Siqueira et al., 2021). However, available data on whether it can be generalized to BAV-AS patients, following a self-expandable TAVR, are conflicting. Several studies (Liao et al., 2018; Forrest et al., 2020) investigating TAVR performance in BAV-AS patients showed comparable valve performance results, but a loss to the follow-up rate of 40%–50% raised a concern about selection bias. Evolut R was used to compare propensity-matched 1-year outcomes after TAVR (Deeb et al., 2022) between different valve morphologies and showed that despite BAV having a lower transvalvular gradient at 30 days after implantation than TAV, no significant difference was observed at the 1-year follow-up between the two groups, indicating a modest and transient trend of a haemodynamic change in the BAV. No significant reduction in PVL was shown according to subgroup analyses (Mangieri et al., 2020) of BAV patients treated with transcatheter self-expandable valves based on the BEAT international collaborative registry. Since the prevalence of the BAV phenotype has been expected to inevitably increase as a result of increased evidence of TAVR in AS patients with lower surgical risks and younger age (Vincent et al., 2021), further exploration of the impact of BAV anatomies on PVL progression after implantation would be of great importance to further facilitate the TAVR application in that population.

Therefore, in this study, we aimed to perform inter- and intra-group analyses of changes in PVL severity, along with AV haemodynamics, following self-expandable TAVR between different aortic valve morphologies to identify the differences.

## Methods

### Patient selection

From January 2016 to August 2019, consecutive patients with severe calcific AS undergoing self-expandable TAVR at our institution were reviewed. Among them, subjects who underwent serial echocardiography and clinical follow-up at 1 and 12 months after valve implantation were finally enrolled. Exclusion criteria included a history of prosthetic degeneration, incidence of periprocedural mortality, and conversion to a surgical treatment.

TAVR procedures were conducted under general anaesthesia by fluoroscopy, as well as transesophageal echocardiography (TEE), as described in full detail elsewhere (Zhou et al., 2020). An informed consent was obtained from each subject, and our study was approved by the Institutional Review Board of Zhongshan Hospital, Fudan University, Shanghai, China (B2019-093).

### Multidetector computed tomography and a sizing strategy

**FIGURE 1:**
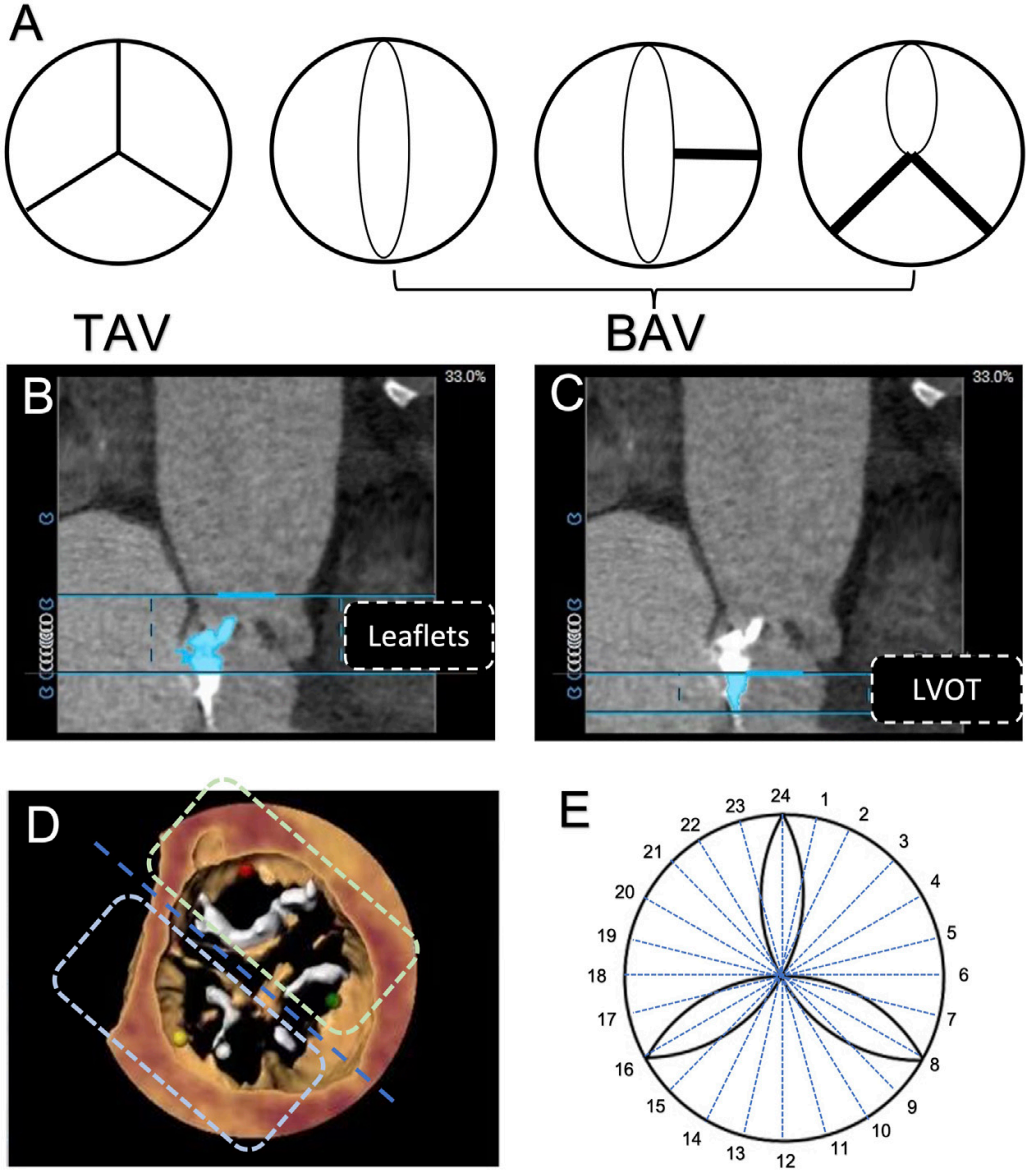
Aortic valve phenotype and calcification evaluation based on computerized tomography. **(A)** Classification of aortic valve morphology. **(B)** Region of the aortic valve leaflet. **(C)** Region of the left ventricular outflow tract. **(D)** Assessment of aortic valve calcification eccentricity by the use of the ‘bi-plane method’. **(E)** 24 sectors were divided by every 15° to calculate the maximum difference of calcification eccentricity.

All patients underwent preoperative evaluation of aortic valves and an aortic root with a 320-detector row computed tomography scanner (Aquilion ONE, Canon Medical Systems, Tochigi-ken, Japan), with a collimation of 160 mm × 0.5 mm, a gantry rotation time of 350 ms, and a high-pitch spiral data acquisition mode. The tube voltage was 100 kV–120 kV, and the tube current was 320 mA–350 mA. The amount of the non-ionic iodinated contrast medium for MDCT examination was 60 ml–80 ml (Ultravist 370, Bayer Schering Pharma, Berlin, Germany) at a flow rate of 4 ml/s–5 ml/s. A saline flush of 25 ml was administered at the same flow rate after iodinated contrast medium administration. To synchronize the arrival of the contrast medium and the scan, bolus arrival was detected by automated peak enhancement detection in the descending aorta with a threshold of 180 Hounsfield units (HU). The total amount of contrast used was dependent on the total scan time and body weight. Data acquisition was performed gated to the electrocardiogram to allow retrospective gating and reconstruction of the data at desired phases of the cardiac cycle (at each 5% RR interval and at 30%–40% for systole and 70%–80% for diastole). Reconstructions of the aortic root were created by 3mensio software (Pie Medical Imaging, Bilthoven, Netherlands), as we previously described (Khalique et al., 2014), and the slide thickness was set as 0.5 mm. All CT images were then independently reviewed by two experienced cardiologists in our centre to attain an interobserver agreement. Our study population was classified into two subgroups: BAV (congenitally malformed valve of two cusps with/without raphae) and TAV (Figure 1A) (Sievers and Schmidtke, 2007). The quantity and distribution of calcification were analysed by using calcium volume (CV) measurements with an empirical starting threshold of 850 HU based on the contrast-enhanced images (Jilaihawi et al., 2014). The total volume of aortic valve calcification was then evaluated and further separated into two regions along the double oblique long axis of the left aortic annulus and left ventricular outflow tract (LVOT): aortic valve leaflets (from the annular plane to each cuspid tip) (Figure 1B) and LVOT (from the basal annular plane to 5 mm below the left ventricle) (Delgado et al., 2011) (Figure 1C). The ‘bipartition method’ was used to calculate the eccentricity of calcium in our study population, as previously described. In brief, an imaginary ‘cutting line’ was drawn across the centre of AV cusps to divide the horizontal plane into two sectors (Figure 1D). Twelve sets of two sectors were generated by rotating the cutting line in 15-degree intervals, and the highest absolute difference in the CV burden (△CV) between the adjacent two sectors was collected (Figure 1E).

Our selection strategy for the valve size was commonly based on the annulus dimensions of the individual image reconstructions in the end-systolic phase (35% of the cardiac cycle), while the nominal thresholds for each size were derived from vendor recommendations. Undersizing (defined as a smaller prosthesis as opposed to the preprocedural CT-predicted size) would be considered in the case of those with severe calcification in the sinus of Valsalva, LVOT, and commissure fusion and evaluated at high risks of coronary obstruction and pacemaker implantation.

### Clinical and echocardiographic data collection during hospitalisation and at follow-up

Baseline demographic and procedure-related data, including the device type, number of implanted valves, performance of balloon post-dilation, permanent pacemaker implantation, and other adverse clinical events, were collected for each subject. At the time of hospital discharge, each patient underwent a transthoracic echocardiographic assessment using an iE33 ultrasound system (Philips Medical System, Best, Netherlands) equipped with an S5-1 transthoracic transducer. The severity of paravalvular leak (PVL) was categorised based on its circumferential extent (sum of the circumferential lengths of each regurgitant jet vena contracta/the circumference of the outer edge of the transcatheter valve) as the VARC-3 criteria suggested (Varc-3 Writing et al., 2021): 1) mild: less than 10%; 2) moderate: between 10% and 30%; and 3) severe: more than 30%. The minimum and maximum diameters of the stent were recorded in the parasternal short-axis view at a mid-to-late diastolic frame, and the stent frame eccentricity index was calculated as 100 × [1 – (minimum stent frame diameter/maximum stent frame diameter)] (Di Martino et al., 2017).

After discharge, a routine transthoracic echocardiogram, along with a clinical evaluation, was performed for each subject at 1 and 12 months and then yearly, following TAVR. Echocardiographic parameters, including the left ventricular ejection fraction (LVEF), aortic valve area, peak velocity, mean transvalvular gradient, and severity of PVL, were assessed and collected. After classifying patients with at least mild PVL into two groups according to the PVL reduction grade at the 1-year follow-up after implantation, the composite endpoint of our present study was then defined as a combination of an all-cause mortality and rehospitalisation for heart failure (HF) (defined as any event requiring oral and/or intravenous therapy) in the next 2 years.

### Statistical analysis

Continuous data were described as the mean ± SD and compared using *t*-test analysis (one-way ANOVA among three groups) or the median (interquartile range) and compared using the Wilcoxon rank-sum test (Kruskal–Wallis test among three groups). Categorical variables were reported as frequencies (percentages), and the chi-squared test or Fisher’s exact test was used for comparison. Clinical outcomes in our study were calculated through Kaplan‒Meier survival analysis and compared according to the log-rank test. Univariate Cox regression models were then used to identify predictors of adverse clinical outcomes during follow-ups, and variables with *p* < 0.1 were considered eligible to be included in the multivariate Cox analysis.

All statistical analyses were performed by SPSS software version 26.0. A two-sided *p*-value of <0.05 was considered to be statistically significant.

## Results

**FIGURE 2:**
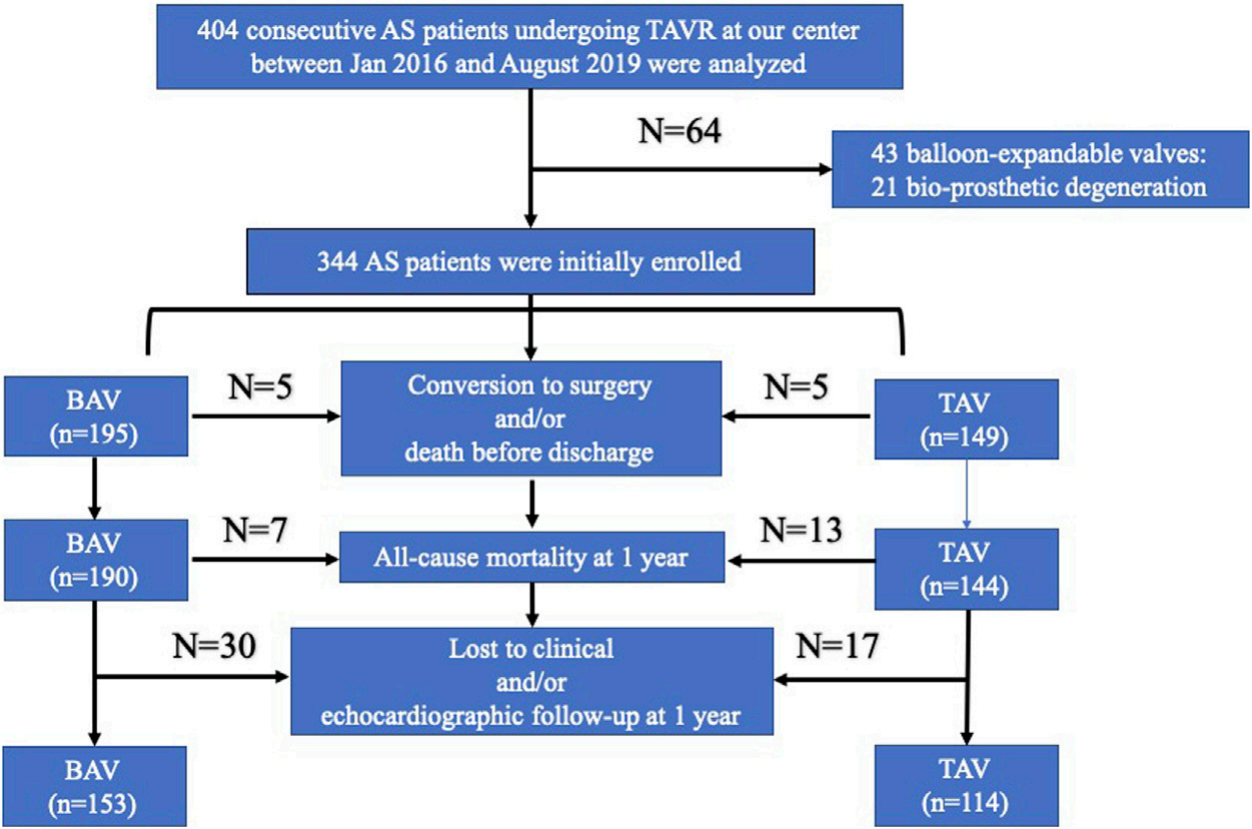
Flowchart of patient selection. BAV: bicuspid aortic valve; TAV: tricuspid aortic valve; TAVR: transcatheter aortic valve replacement; AS: aortic stenosis.

A total of 267 AS patients undergoing TAVR were finally enrolled in our study, including 153 BAV and 114 TAV patients (Figure 2). The demographic and clinical characteristics are shown in Table 1. Advanced age (74.63 ± 7.67 *vs.* 77.37 ± 6.92, y = 0.003) and more cases of hypertension (43.14% *vs.* 74.56%, *p* < 0.001) were found in the BAV group, while the mean STS score (4.54 ± 1.86 vs. 4.68 ± 1.58, *p* = 0.512) was comparable between the two groups. Regarding procedure-related details, no significant difference was found in the incidence of a second valve implantation (7.19% *vs.* 3.51%, *p* = 0.283), transcatheter valve device (*p* = 0.964), or new permanent pacemaker implantation (21.57% *vs.* 14.91%, *p* = 0.978). A higher prevalence of undersizing (35.95 *vs.* 11.40%, *p* < 0.001), as well as post-dilation (37.25% *vs.* 25.46%, *p* = 0.028), was observed in the BAV group.

**TABLE 1:** Baseline characteristics.

	BAV (*n* = 153)	TAV (*n* = 114)	*P* value
Age (yrs)	74.63 ± 7.67	77.37 ± 6.92	0.003
Sex, male (n,%)	55 (35.95)	54 (47.37)	0.060
BMI	23.33 ± 2.94	23.92 ± 3.69	0.185
STS score	4.54 ± 1.86	4.68 ± 1.58	0.512
NYHA class (n,%)			0.497
II	27 (17.65)	25 (21.93)	
III	98 (64.04)	65 (57.02)	
IV	28 (18.30)	24 (21.05)	
Hypertension (n,%)	66 (43.14)	85 (74.56)	<0.001
Diabetes (n,%)	26 (16.99)	30 (26.32)	0.064
CKD (n,%)	15 (9.80)	20 (17.54)	0.061
Coronary artery disease (n,%)	10 (6.54)	7 (6.14)	0.896
Stroke/TIA (n,%)	7 (4.58)	4 (3.51)	0.763
Previous CABG (n,%)	0	2 (1.75)	0.181
Previous PCI (n,%)	10 (6.54)	15 (13.16)	0.066
Baseline echocardiographic parameters			
LVEF (%)	56.69 ± 11.95	58.43 ± 11.19	0.234
SPAP (mmHg)	36.71 ± 5.96	34.89 ± 6.70	
Peak velocity (m/s)	4.84 ± 0.76	4.65 ± 0.75	0.331
Mean gradient (mmHg)	56.58 ± 18.33	53.09 ± 16.88	0.118
Aortic regurgitation (n,%)			0.008
None	126 (82.35)	75 (65.79)	
Mild	20 (13.07)	30 (26.32)	
≥Moderate	7 (4.58)	9 (7.89)	
≥Moderate mitral regurgitation (n,%)	18 (11.76)	15 (13.16)	0.732
CT-derived annular parameters			
Perimeter (mm)	83.68 ± 8.55	81.50 ± 7.16	0.228
Area (mm²)	502 (409.50–589.25)	464.50 (403.00–523.75)	0.068
Aortic valve calcification (mm³)			
Leaflet CV_total_	557.90 (321.50–884.00)	415.10 (199.40–714.70)	<0.001
△Leaflet CV	173.70 (86.00–321.41)	133.00 (75.10–224.70)	0.043
LVOT CV_total_	0 (0–33.48)	0 (0–23.38)	0.334
△LVOT CV	0 (0–13.3)	0 (0–9.40)	0.555
Procedural characteristics			
Device (n,%)			0.964
Venus-A	89 (58.17)	66 (57.89)	
Vita-flow	64 (41.83)	48 (42.11)	
Undersizing (n,%)	55 (35.95)	13 (11.40)	<0.001
Implantation of two valves (n,%)	11 (7.19)	4 (3.51)	0.283
Permanent pacemaker implantation (n,%)	23 (21.57)	17 (14.91)	0.978
Balloon post-dilation (n,%)	57 (37.25)	28 (25.46)	0.028

BAV: bicuspid aortic valve; TAV: tricuspid aortic valve; NYHA: New York Heart Association; CKD: chronic kidney disease; TIA: transient ischemic attack; CABG: coronary artery bypass grafting; PCI: percutaneous coronary artery intervention; LVEF: left ventricular ejection fraction; SPAP: systolic pulmonary artery pressure; CT: computed tomography.

### Echocardiographic parameters following TAVR

A summary of the echocardiographic findings for the entire study population at baseline, hospital discharge, and 1 month and 1 year following TAVR is shown in Table 2.

**TABLE 2:** Haemodynamics before discharge and during follow-ups.

Variable	BAV (*n* = 153)	TAV (*n* = 114)	*p*-value
Before discharge			
LVEF (%)	58.73 ± 9.24	60.27 ± 8.93	0.190
SPAP (mmHg)	36.71 ± 5.98	34.89 ± 6.70	0.486
Peak velocity (m/s)	2.19 ± 0.52	2.10 ± 0.61	0.188
Mean gradient (mmHg)	11.63 ± 5.55	10.66 ± 5.28	0.149
EOA (mm^2^)	2.17 ± 0.59	2.18 ± 0.55	0.831
1-month follow-up			
LVEF (%)	59.72 ± 8.80	61.04 ± 7.10	0.193
SPAP (mmHg)	37.47 ± 11.50	36.65 ± 8.37	0.761
Peak velocity (m/s)	2.04 ± 0.52	1.99 ± 0.55	0.439
Mean gradient (mmHg)	10.07 ± 5.45	9.82 ± 4.30	0.681
EOA (mm^2^)	2.27 ± 0.65	2.25 ± 0.51	0.884
12-month follow-up			
LVEF (%)	60.20 ± 7.86	61.12 ± 6.80	0.732
SPAP (mmHg)	35.32 ± 9.89	34.18 ± 12.81	0.557
Peak velocity (m/s)	2.00 ± 0.50	1.90 ± 0.53	0.130
Mean gradient (mmHg)	10.03 ± 4.85	9.11 ± 4.03	0.103
EOA (mm^2^)	2.32 ± 0.54	2.32 ± 0.52	0.951

EOA, effective orifice area; other abbreviations are shown as mentioned previously.

**FIGURE 3:**
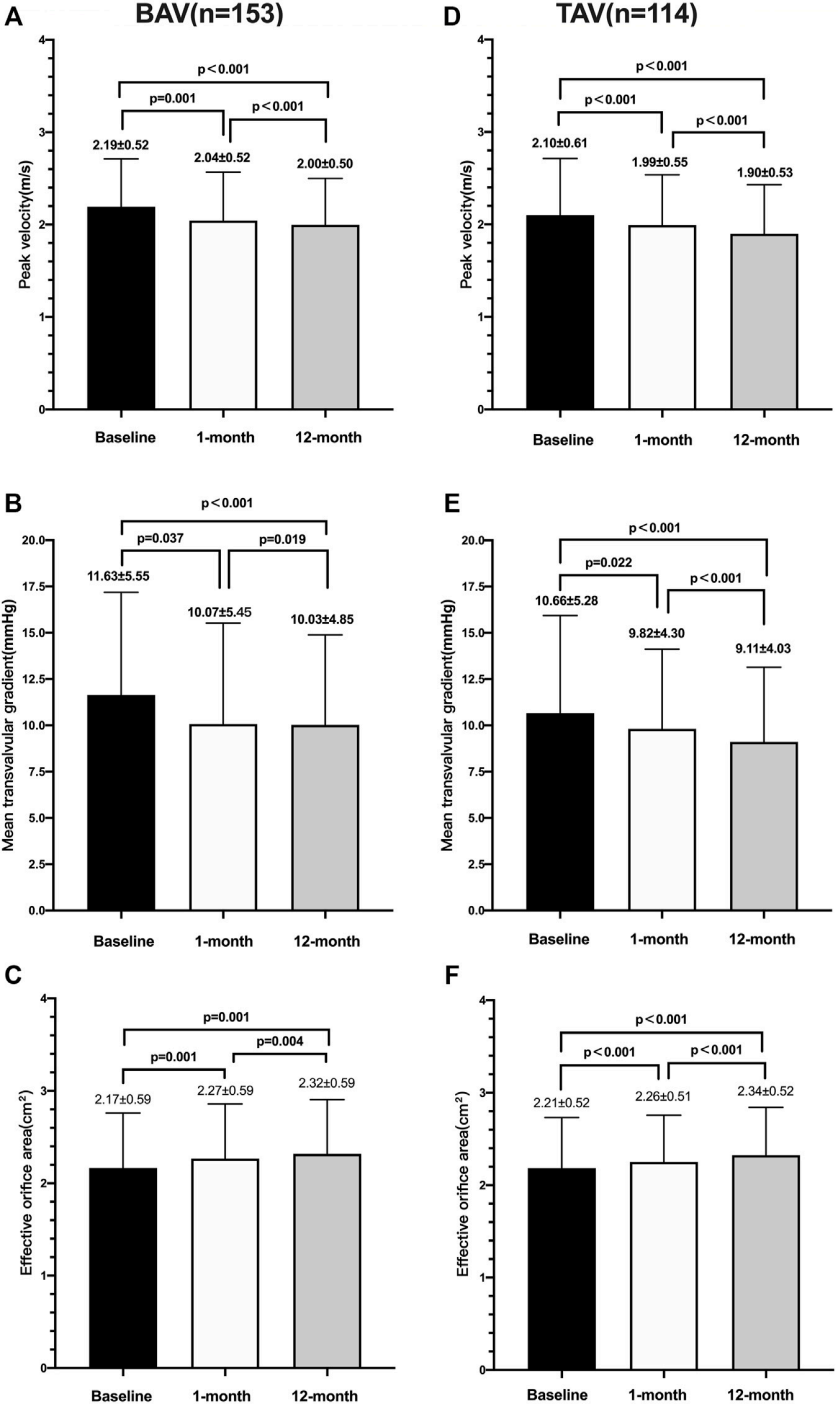
Echocardiographic parameters over time between the BAV and TAV. Changes of peak velocity **(A)**, mean transvalvular gradient **(B)**, and effective orifice area (mm^2^) **(C)** in patients with the bicuspid aortic valve; changes of peak velocity **(D)** mean transvalvular gradient **(E)**, and effective orifice area (mm^2^) **(F)** in patients with the tricuspid aortic valve.

Before discharge, no significant difference was found in the peak velocity (2.19 ± 0.52 m/s *vs.* 2.10 m/s ± 0.61 m/s, *p* = 0.188), mean gradient (11.63 mmHg ± 5.55 mmHg *vs.* 10.66 mmHg ± 5.28 mmHg, *p* = 0.149), or EOA (2.17 ± 0.59 mm^2^ vs. 2.18 ± 0.55 mm^2^, p = 0.831) between the two groups. According to the intragroup analysis based on the paired cohort (Figure 3), statistically significant reductions in the peak velocity, mean transvalvular gradient, and EOA from discharge to the 1-month follow-up were observed in both the BAV (*p* < 0.05) and TAV (*p* < 0.05) groups, and a decreased tendency was maintained until the 1-year follow-up (*p* < 0.001).

### Severity of PVL after implantation

The overall severity of paravalvular regurgitation at the follow-up is presented in Table 3. Of our entire study population, the rates of paravalvular leakage were none in 66.67% (102/153), mild in 24.18% (37/153), and moderate in 9.15% (14/153) for the BAV, and none in 63.16% (72/114), mild in 32.46% (37/114), and moderate in 4.39% (5/114) for the TAV, following TAVR. In the self-matched dataset of the paired cohort, BAV did not show a significant difference in the proportion of PVL severity during follow-ups, but for the TAV group, a notable improvement for PVL was present at the 1-year follow-up *versus* at discharge. More specifically, 14 of 153 (9.15%) BAV patients showed improvements in PVL by at least one grade, including 10 subjects decreasing from mild PVL to none and four cases reducing from moderate to mild PVL (Supplementary Table S1). In the TAV group, 21 of 114 (18.42%) showed improvements in PVL by at least one grade, including 18 subjects decreasing from mild PVL to none. Also, three cases reduced from moderate PVL to mild (*n* = 1) and none (*n* = 2) (Supplementary Table S2). For intergroup comparison, the overall difference in PVL severity did not reach a final significant difference at the 1-year follow-up, but a lower moderate paravalvular regurgitation could be observed in the TAV (*p* = 0.041).

**TABLE 3:** Severity of PVL at 1 month and 12 months after discharge.

	PVL	Before discharge	1 month	12 month	*p*-value		
					Before discharge vs. 1 month	1-month vs. 12 month	Before discharge vs. 12 month
BAV (*n* = 153)					0.652	0.568	0.765
	None	102 (66.67)	99 (64.71)	107 (69.93)	—	—	—
	Mild	37 (24.18)	43 (28.10)	35 (22.88)	0.178	0.330	0.539
	Moderate	14 (9.15)	11 (7.19)	11 (7.19)			
TAV (*n* = 114)					0.438	0.180	0.029
	None	72 (63.16)	78 (68.42)	90 (78.95)	—	—	—
	Mild	37 (32.46)	34 (29.82)	22 (19.30)	0.402	0.071	0.009
	Moderate	5 (4.39)	2 (1.76)	2 (1.75)			
*p*-value (BAV *vs.* TAV)	Overall	0.148	0.125	0.079	—	—	—
	≥mild	0.552	0.668	0.098	—	—	—
	≥moderate	0.134	0.041	0.041	—	—	—

PVL, paravalvular leak; other abbreviations are shown as previously mentioned.

### Predictors for PVL reduction <1 grade within 1 year in patients with at least mild PVL following TAVR

The baseline characteristics and periprocedural outcomes of patients with at least mild PVL at discharge are shown in Supplementary Table S3 and Table 4. According to the univariate logistic regression analysis, BAV, △leaflet CV and eccentric index of the prosthetic valve, and undersizing were found to be associated with PVL reduction <1 grade (*p* < 0.05). Multivariate analysis showed that only BAV (OR: 6.525, 95% CI: 1.462–29.119, and *p* = 0.016), △leaflet CV, per 10 mm³ increase (OR: 1.287, 95% CI: 1.156–0.011, and *p* < 0.001), and undersizing (OR: 4.132, 95% CI: 1.036–16.480, and *p* = 0.044) were still confirmed as independent predictors of △PVL <1 grade at the 1-year follow-up (Table 4). The Hosmer–Lemeshow goodness-of-fit *p*-value was 0.359, and the C-statistic was 0.747, thus confirming good calibration and discrimination of the multivariate model.

**TABLE 4:** Independent predictors for the failure of improvement in PVL by lower than one grade at a 1-year follow-up after TAVR.

Variable	Univariate			Multivariate		
	OR	95% CI	*p*-value	OR	95% CI	*p*-value
Age	0.962	0.906–1.021	0.202	—	—	—
Male	0.795	0.324–1.951	0.616	—	—	—
BMI	0.891	0.778–1.020	0.096	—	—	—
Hypertension	0.515	0.218–1.214	0.129	—	—	—
STS	0.958	0.746–1.230	0.734	—	—	—
BAV	2.643	1.115–6.262	0.027	6.525	1.462–29.119	0.016
△Leaflet CV, average increase 10 mm³	1.230	1.132–1.337	<0.001	1.287	1.156–3.433	<0.001
Prosthetic valve (Venus A *vs.* VitaFlow)	0.622	0.258–1.502	0.291	—	—	—
Undersizing	6.087	2.282–16.238	<0.001	4.132	1.036–16.480	0.044
Valve in valve	0.873	0.138–5.503	0.885	—	—	—
PPM	1.200	0.333–4.328	0.781	—	—	—
Moderate PVL at discharge	1.894	0.894–4.012	0.095	—	—	—
Eccentricity index	27.43	16.28–45.44	0.003	—	—	—
△LVEF	0.976	0.915–1.047	0.536	—	—	—

Abbreviations are shown as mentioned previously.

### Comparison of long-term clinical outcomes between patients (≥mild PVL before discharge) with PVL reduction ≥1 grade and <1 grade

**FIGURE 4:**
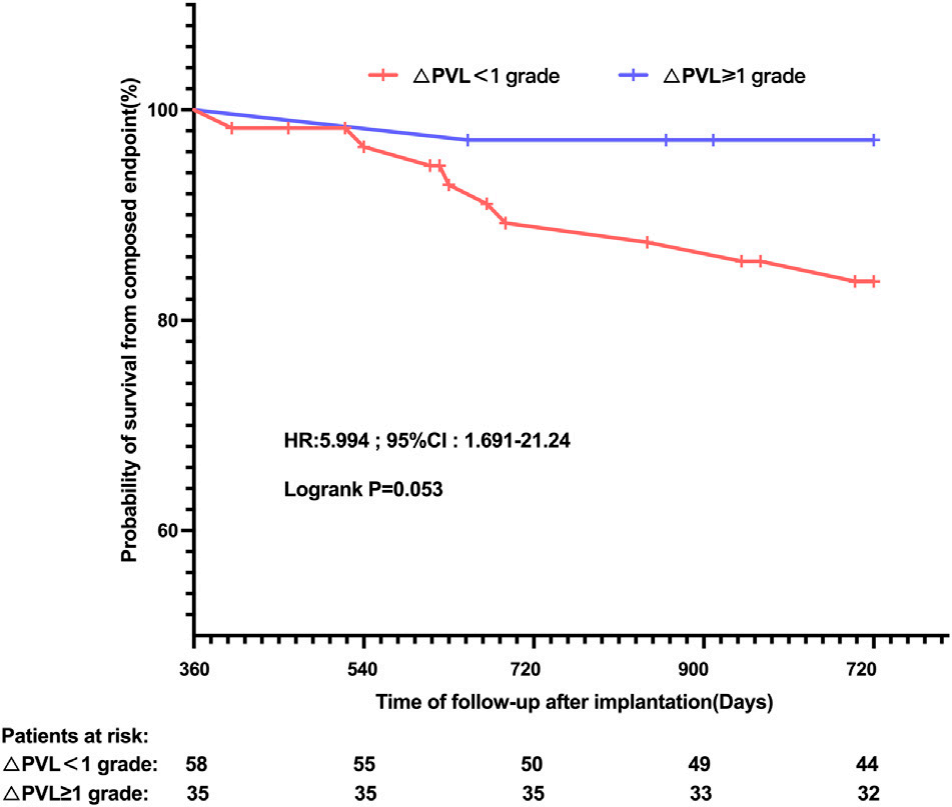
Kaplan–Meier estimates of the rate of the primary composite endpoint according to the PVL reduction grade at 1-year follow-up, following TAVR, in patients with at least mild PVL before discharge. PVL: paravalvular leak.

Among patients with at least mild PVL following TAVR, 3/35 (8.57%) in subgroups with PVL reduction <+1 and 4/58 (6.90%) in subgroups with PVL reduction ≥+1 were lost to the 3-year follow-up after implantation. Taking the individual’s 1-year follow-up point as the starting point for the Kaplan–Meier analysis in our study (Figure 4), a trend towards a higher risk of all-cause mortality and cardiovascular-related rehospitalisation at the 3-year follow-up could be observed in the BAV group (83.70% *vs.* 97.14% and log-rank *p* = 0.053) in comparison with the TAV group.

## Discussion

To the best of our knowledge, our study provides a preliminary analysis to explore the difference in PVL regression and haemodynamics between SEVs implanted in the BAV and TAV groups along with its related factors. The main findings of our study could be summarized as follows: 1) continuous improvement of self-expandable valve haemodynamics could be observed in both the BAV and TAV groups during follow-ups; 2) PVL regression was more prevalent in the TAV group than in the BAV group at the 1-year follow-up after TAVR; 3) the native BAV phenotype, asymmetric valve leaflet calcification, and undersizing were independent predictors for PVL reduction <1 grade.

A previous patient-specific numerical study (Li et al., 2022) showed that lower stress on the aortic valve was generated by SEVs than in BEVs; hence, the self-expandable stent was more susceptible to the surrounding calcium and had a higher risk of representing as an ellipse immediately after implantation, resulting in a considerable risk of PVL. However, considering its continuous outwarding force, the basic state of the nitinol frame was on a gradual reformation progress and could be closer to the aortic annulus on the beating heart. An improvement of prosthetic valve haemodynamics compared with discharge following TAVR has been reported in previous studies (Blackman et al., 2019; Abdel-Wahab et al., 2020). In the present study, we also indicated that for TAV patients undergoing TAVR with either the Venus A or VitaFlow valve, a marked improvement in valve haemodynamics was noted 1 month after implantation and could be maintained until the 1-year follow-up. For the BAV, statistically significant improvements in the aortic valve velocity, mean gradient, and effective orifice area were also detected. Intergroup analyses showed that these echocardiographic variables were all comparable during a follow-up between the two groups, indicating that a similar trend towards the gradual self-expansion of the bioprosthesis also occurred in BAV patients receiving a self-expandable TAVR.

However, conflicting results of PVL regression in different aortic valve morphologies were indicated in our study. Findings from the CoreValve U.S. Pivotal Trial (Oh et al., 2015) reported a significant reduction in PVL over 1 year of follow-ups in TAV patients. A similar improvement in PVL severity after discharge in TAV patients was also shown in our study, but these subjects with native bicuspid valves failed. Our analysis of paired PVL data in two cohorts reported that the BAV was associated with a significantly higher prevalence of PVL regression <1 grade in patients with at least mild PVL, following TAVR. Considering that tissue ingrowth covering the paravalvular space was an early phenomenon after implantation (Rallidis et al., 1999), a plausible explanation for our long-standing difference might be the lower reshaping efficiency of the basic non-circular prosthetic geometry in the BAV. Anatomical abnormalities, including the elliptical annulus, asymmetrical aortic cusps, bulky but friable calcium, and eccentric calcification, have been acknowledged to commonly coexist with bicuspid valves, significantly influencing the shape of the prosthetic frame (Xiong et al., 2022). In our study, the stent eccentricity index according to the periprocedural three-dimensional oesophago echocardiographic assessment was much higher in the BAV group. SEVs can hardly be re-established into a circular cross-section shape, depending solely on the interaction of an inner-expansion force and cardiac contraction (Qiu et al., 2021). On the other hand, a higher placement of the valve within the aortic annulus and undersizing device selection were, therefore, more prevalent in the BAV. Hence, the continuous expansion force of the stent itself might be insufficient to regress or close the paravalvular gaps between the nitinol frame and the native aortic valve tissue, especially once the native aortic annulus was not stretched enough. The multivariate logistic analysis in our study demonstrated that the BAV, asymmetric calcification distribution, and undersizing choice could predict the failure of PVL regression after TAVR. This provided statistical support for our hypothesis. Another possible contributing factor to our conflicting data in PVL reduction between different aortic valve morphologies might be the numerically higher incidence of post-dilation in the BAV. Although no significant difference was reached, post-dilation, to some extent, exerted an external force to have a circular deployment stent shape. In the case of BAV patients’ unresponsiveness to post-dilation, there could be little prospect of dramatic reformation, relying on the inner expansion itself afterwards.

The severity of PVL remains a serious challenge affecting the long-term prognosis after TAVR (Genereux et al., 2013; Terre et al., 2017; Avvedimento and Tang, 2021). In the present study, from a new perspective, we reported a potent positive relationship between the failure of PVL regression and higher risk of adverse cardiovascular events during a mid-to-long-term clinical follow-up. This was consistent with previous findings (Giblett et al., 2019; Giblett et al., 2022) that residual PVL would result in persistent heavier cardiac preload and excessive consumption of heart compensatory mechanisms. Given that patients with at least mild PVL at discharge and significant PVL reduction at the 1-year follow-up might harbour a 6.0-fold increase in the subsequent 2-year poor prognosis, our studies intended to raise a concern about the easy generalization of TAVR to the BAV-AS population, especially in the case of those evaluated at a high risk of paravalvular regurgitation at preprocedural planning.

## Limitations

Our study was retrospectively performed and derived from a single-centre experience with a relatively small sample size. The identical inclusion of patients fulfilling clinical and echocardiographic follow-ups after TAVR could result in considerable selection bias and confounding bias that might influence the observed results. Another important concern should be raised regarding the lack of detailed data for the BAV subtypes enrolled in our study. It has been acknowledged that different BAV characteristics (raphae, especially calcified, and excess and asymmetric calcium burden) could significantly affect TAVR prognosis. Further studies are needed to further discuss the impact of different BAV morphologies on PVL regression upon a longer term follow-up. Moreover, only the first-generation SEVs were involved in our present study; thus, our findings could not be generalized to the new-generation prostheses. Finally, a lack of CT data after TAVR during the mid-to-long term follow-up was observed in our study, and further investigation of stent reformation might facilitate interpretation of our findings of differences in PVL regression between different valve morphologies.

## Conclusion

Further AV haemodynamic improvement compared with the discharge existed, following the self-expandable TAVR, regardless of aortic valve morphology. However, regression of PVL over time, up to 1 year, was undesirable in AS patients with a native BAV, and asymmetric calcification and the use of an undersized prosthesis and failure of PVL regression were associated with impaired long-term clinical prognosis.

## Data Availability

The original contributions presented in the study are included in the article/Supplementary Material; further inquiries can be directed to the corresponding author.

## References

[B1] Abdel-Wahab M. Mehilli J. Frerker C. Neumann F. J. Kurz T. Tolg R. (2014). Comparison of balloon-expandable vs self-expandable valves in patients undergoing transcatheter aortic valve replacement: the CHOICE randomized clinical trial. JAMA 311, 1503–1514. 10.1001/jama.2014.3316 24682026

[B2] Abdel-Wahab M. Landt M. Neumann F. J. Massberg S. Frerker C. Kurz T. (2020). 5-Year outcomes after TAVR with balloon-expandable versus self-expanding valves: Results from the CHOICE randomized clinical trial. JACC. Cardiovasc. Interv. 13, 1071–1082. 10.1016/j.jcin.2019.12.026 32305398

[B3] Avvedimento M. Tang G. H. L. (2021). Transcatheter aortic valve replacement (TAVR): Recent updates. Prog. Cardiovasc. Dis. 69, 73–83. 10.1016/j.pcad.2021.11.003 34800439

[B4] Blackman D. J. Saraf S. MacCarthy P. A. Myat A. Anderson S. G. Malkin C. J. (2019). Long-term durability of transcatheter aortic valve prostheses. J. Am. Coll. Cardiol. 73, 537–545. 10.1016/j.jacc.2018.10.078 30732706

[B5] Deeb G. M. Reardon M. J. Ramlawi B. Yakubov S. J. Chetcuti S. J. Kleiman N. S. (2022). Propensity-matched 1-year outcomes following transcatheter aortic valve replacement in low-risk bicuspid and tricuspid patients. JACC. Cardiovasc. Interv. 15, 511–522. 10.1016/j.jcin.2021.10.027 35272776

[B6] Deharo P. Bisson A. Herbert J. Lacour T. Saint Etienne C. Grammatico-Guillon L. (2020). Impact of sapien 3 balloon-expandable versus evolut R self-expandable transcatheter aortic valve implantation in patients with aortic stenosis: data from a nationwide analysis. Circulation 141, 260–268. 10.1161/CIRCULATIONAHA.119.043971 31736332

[B7] Delgado V. Ng A. C. T. Schuijf J. D. van der Kley F. Shanks M. Tops L. F. (2011). Automated assessment of the aortic root dimensions with multidetector row computed tomography. Ann. Thorac. Surg. 91, 716–723. 10.1016/j.athoracsur.2010.09.060 21352985

[B8] Di Martino L. F. M. Soliman O. I. I. van Gils L. Vletter W. B. Van Mieghem N. M. Ren B. (2017). Relation between calcium burden, echocardiographic stent frame eccentricity and paravalvular leakage after corevalve transcatheter aortic valve implantation. Eur. Heart J. Cardiovasc. Imaging 18, 648–653. 10.1093/ehjci/jex009 28369281

[B9] Forrest J. K. Kaple R. K. Ramlawi B. Gleason T. G. Meduri C. U. Yakubov S. J. (2020). Transcatheter aortic valve replacement in bicuspid versus tricuspid aortic valves from the STS/ACC TVT registry. JACC. Cardiovasc. Interv. 13, 1749–1759. 10.1016/j.jcin.2020.03.022 32473890

[B10] Genereux P. Head S. J. Hahn R. Daneault B. Kodali S. Williams M. R. (2013). Paravalvular leak after transcatheter aortic valve replacement: the new achilles' heel? A comprehensive review of the literature. J. Am. Coll. Cardiol. 61, 1125–1136. 10.1016/j.jacc.2012.08.1039 23375925

[B11] Giblett J. P. Rana B. S. Shapiro L. M. Calvert P. A. (2019). Percutaneous management of paravalvular leaks. Nat. Rev. Cardiol. 16, 275–285. 10.1038/s41569-018-0147-0 30659248

[B12] Giblett J. P. Williams L. K. Moorjani N. Calvert P. A. (2022). Percutaneous management of paravalvular leaks. Heart 108, 1005–1011. 10.1136/heartjnl-2021-319159 34686568

[B13] Jilaihawi H. Makkar R. R. Kashif M. Okuyama K. Chakravarty T. Shiota T. (2014). A revised methodology for aortic-valvar complex calcium quantification for transcatheter aortic valve implantation. Eur. Heart J. Cardiovasc. Imaging 15, 1324–1332. 10.1093/ehjci/jeu162 25187618

[B14] Kaya D. Tanriverdi Z. Dursun H. Colluoglu T. (2016). Echocardiographic outcomes of self-expandable CoreValve versus balloon-expandable edwards SAPIEN XT valves: the comparison of two bioprosthesis implanted in a single centre. Int. J. Cardiovasc. Imaging 32, 1371–1378. 10.1007/s10554-016-0924-y 27278270

[B15] Khalique O. K. Hahn R. T. Gada H. Nazif T. M. Vahl T. P. George I. (2014). Quantity and location of aortic valve complex calcification predicts severity and location of paravalvular regurgitation and frequency of post-dilation after balloon-expandable transcatheter aortic valve replacement. JACC. Cardiovasc. Interv. 7, 885–894. 10.1016/j.jcin.2014.03.007 25147034

[B16] Lanz J. Kim W-K. Walther T. Burgdorf C. Möllmann H. Linke A. (2019). Safety and efficacy of a self-expanding versus a balloon-expandable bioprosthesis for transcatheter aortic valve replacement in patients with symptomatic severe aortic stenosis: a randomised non-inferiority trial. Lancet 394, 1619–1628. 10.1016/S0140-6736(19)32220-2 31570258

[B17] Li J. Yan W. Wang W. Wang S. Wei L. (2022). Comparison of balloon-expandable valve and self-expandable valve in transcatheter aortic valve replacement: A patient-specific numerical study. J. Biomech. Eng. 144, 104501. 10.1115/1.4054332 35420119

[B18] Liao Y. B. Li Y. J. Xiong T. Y. Ou Y. W. Lv W. Y. He J. L. (2018). Comparison of procedural, clinical and valve performance results of transcatheter aortic valve replacement in patients with bicuspid versus tricuspid aortic stenosis. Int. J. Cardiol. 254, 69–74. 10.1016/j.ijcard.2017.12.013 29246428

[B19] Mangieri A. Tchetche D. Kim W. K. Pagnesi M. Sinning J. M. Landes U. (2020). Balloon versus self-expandable valve for the treatment of bicuspid aortic valve stenosis: Insights from the BEAT international collaborative registrys. Circ. Cardiovasc. Interv. 13, e008714. 10.1161/CIRCINTERVENTIONS.119.008714 32646304

[B20] Oh J. K. Little S. H. Abdelmoneim S. S. Reardon M. J. Kleiman N. S. Lin G. (2015). Regression of paravalvular aortic regurgitation and remodeling of self-expanding transcatheter aortic valve: an observation from the CoreValve U.S. Pivotal trial. JACC. Cardiovasc. Imaging 8, 1364–1375. 10.1016/j.jcmg.2015.07.012 26508386

[B21] Popma J. J. Deeb G. M. Yakubov S. J. Mumtaz M. Gada H. O'Hair D. (2019). Transcatheter aortic-valve replacement with a self-expanding valve in low-risk patients. N. Engl. J. Med. 380, 1706–1715. 10.1056/NEJMoa1816885 30883053

[B22] Qiu D. Barakat M. Hopkins B. Ravaghi S. Azadani A. N. (2021). Transcatheter aortic valve replacement in bicuspid valves: The synergistic effects of eccentric and incomplete stent deployment. J. Mech. Behav. Biomed. Mat. 121, 104621. 10.1016/j.jmbbm.2021.104621 34130079

[B23] Rallidis L. S. Moyssakis I. E. IkonomIdIs I. NihoyannoPoulos P. (1999). Natural history of early aortic paraprosthetic regurgitation: a five-year follow-up. Am. Heart J. 138, 351–357. 10.1016/s0002-8703(99)70124-9 10426851

[B24] Shishido K. Yamanaka F. Ochiai T. Yamabe T. Noguchi K. Ota T. (2019). Hemodynamic comparison of CoreValve and SAPIEN-XT TAVI valves in Japanese patients. Heart Vessels 34, 1674–1683. 10.1007/s00380-019-01414-0 30993441

[B25] Sievers H. H. Schmidtke C. (2007). A classification system for the bicuspid aortic valve from 304 surgical specimens. J. Thorac. Cardiovasc. Surg. 133, 1226–1233. 10.1016/j.jtcvs.2007.01.039 17467434

[B26] Siqueira D. A. Simonato M. Ramos A. A. Bignoto T. Le Bihan D. Barreto R. B. M. (2021). Mid- to long-term clinical and echocardiographic outcomes after transcatheter aortic valve replacement with a new-generation, self-expandable system. Catheter. Cardiovasc. Interv. 97, 167–174. 10.1002/ccd.28999 32445607

[B27] Sondergaard L. Rodes-Cabau J. Hans-Peter Linke A. Fichtlscherer S. Schafer U. Kuck K. H. (2018). Transcatheter aortic valve replacement with a repositionable self-expanding prosthesis: the PORTICO-I trial 1-year outcomes. J. Am. Coll. Cardiol. 72, 2859–2867. 10.1016/j.jacc.2018.09.014 30261238

[B28] Terre J. A. George I. Smith C. R. (2017). Pros and cons of transcatheter aortic valve implantation (TAVI). Ann. Cardiothorac. Surg. 6, 444–452. 10.21037/acs.2017.09.15 29062739 PMC5639221

[B29] Varc-3 Writing C. Genereux P. Piazza N. Alu M. C. Nazif T. Hahn R. T. (2021). Valve academic research consortium 3: Updated endpoint definitions for aortic valve clinical research. J. Am. Coll. Cardiol. 77, 2717–2746. 10.1016/j.jacc.2021.02.038 33888385

[B30] Vincent F. Ternacle J. Denimal T. Shen M. Redfors B. Delhaye C. (2021). Transcatheter aortic valve replacement in bicuspid aortic valve stenosis. Circulation 143, 1043–1061. 10.1161/CIRCULATIONAHA.120.048048 33683945

[B31] Xiong T. Y. Ali W. B. Feng Y. Hayashida K. Jilaihawi H. Latib A. (2022). Transcatheter aortic valve implantation in patients with bicuspid valve morphology: a roadmap towards standardization. Nat. Rev. Cardiol. 10.1038/s41569-022-00734-5 35726019

[B32] Zhou D. Pan W. Wang J. Wu Y. Chen M. Modine T. (2020). VitaFlow transcatheter valve system in the treatment of severe aortic stenosis: One-year results of a multicenter study. Catheter. Cardiovasc. Interv. 95, 332–338. 10.1002/ccd.28226 31020788

