# Supplementary material for: Aortic valve morphology and paravalvular leak regression after a self-expandable transcatheter aortic valve replacement
Source: Front Physiol. 2023 Jan 6;13:1088681. doi: 10.3389/fphys.2022.1088681 (PMC9853023; doi:10.3389/fphys.2022.1088681)
Supplement: Supplementary file 1 [file DataSheet1.docx]

**Supplementary table 1 Regression of PVL at 1-year follow-up in BAV groups**

| BAV(n= 153) | 1-year follow-up after TAVR | | |
| --- | --- | --- | --- |
| At discharge | None | Mild | Moderate |
| None | 97 | 5 | 0 |
| Mild | 10 | 26 | 1 |
| Moderate | 0 | 4 | 10 |

Green represented improvement in PVL by at least 1 grade;

Red represented worsening in PVL by at least 1 grade.

**Supplementary Table 2 Regression of PVL at 1-year follow-up in TAV groups**

| TAV(n= 114) | 1-year follow-up after TAVR | | |
| --- | --- | --- | --- |
| At discharge | None | Mild | Moderate |
|  |  |  |  |
| None | 70 | 2 | 0 |
| Mild | 18 | 19 | 0 |
| Moderate | 2 | 1 | 2 |

Green represented improvement in PVL by at least 1 grade;

Red represented worsening in PVL by at least 1 grade.

**Supplementary Table 3 Baseline characteristics of TAVR patients with ≥mild PVL before discharge**

|  | △PVL < 1 grade  (n = 58) | △PVL ≥1 grade  (n = 35) | P value |
| --- | --- | --- | --- |
| Age(yrs) | 75.4±7.57 | 77.46±6.68 | 0.203 |
| Male(n,%) | 6(10.34) | 6(17.14) | 0.343 |
| BMI | 22.67±3.23 | 23.88±3.30 | 0.093 |
| STS score | 4.71±1.61 | 4.83±1.81 | 0.737 |
| NYHA≥III(n,%) | 47(81.03) | 30(85.71) | 0.562 |
| Hypertension(n,%) | 27(46.55) | 22(62.86) | 0.127 |
| Diabetes(n,%) | 11(18.97) | 8(2.86) | 0.652 |
| Echocardiographic parameters |  |  |  |
| LVEF(%) | 54.70±11.45 | 56.94±11.70 | 0.378 |
| Peak velocity(m/s) | 4.85±0.65 | 5.21±0.77 | 0.298 |
| Mean gradient(mmHg) | 54.37±16.60 | 59.94±20.27 | 0.158 |
| EOA(cm²) | 0.73±0.15 | 0.67±0.14 | 0.198 |
| ≥moderate AR(n.%) | 6(10.34) | 2(5.71) | 0.706 |
| CT-derived parameters |  |  |  |
| BAV(n,%) | 37(63.79) | 14(40.00) | 0.026 |
| Annulus area(mm²) | 500.00  (426.00-567.00) | 453.00  (390.00-518.00) | 0.228 |
| Annulus perimeter(mm) | 83.67±8.55 | 81.50±7.16 | 0.068 |
| Aortic valve calcification(mm³) |  |  |  |
| Leaflet CV_total_ | 758.75  (372.45-1051.38) | 771.40  (431.20-982.00) | 0.962 |
| △Leaflet CV | 189.50  (91.15-356.25) | 91.50  (51.00-176.00) | 0.016 |
| LVOT CV_total_ | 0(0-49.25) | 0(0-34.10) | 0.711 |
| △LVOT CV | 0(0-45.28) | 0(0-27.40) | 0.981 |

Abbreviations were shown as above.

**Supplementary Table 4 Procedural outcomes and echocardiographic measures for TAVR patients with ≥mild PVL**

|  | △PVL < 1 grade  (n = 58) | △PVL ≥1 grade  (n = 35) | | P value | |
| --- | --- | --- | --- | --- | --- |
| Valve type(n,%) |  |  | | 0.289 | |
| Venus-A | 41(70.69) | 21(60.00) | |  | |
| Vita-flow | 17(29.31) | 14(40.00) | |  | |
| Valve size(n,%) |  |  | | 0.356 | |
| ≤23mm | 9(15.51) | 9(25.71) | |  | |
| >23mm,≤26mm | 29(50.00) | | 17(48.57) | |  |
| >26mm,≤29mm | 16(27.59) | 9(25.71) | |  | |
| >29mm | 4(6.90) | 0 | |  | |
| undersizing (n,%) | 28(48.28) | 7(20.00) | | 0.006 | |
| Post-dilation(n,%) | 26(44.83) | 13(37.14) | | 0.520 | |
| Implantation of two valves (n,%) | 3(5.17) | 2(5.71) | | ≥0.999 | |
| New pacemaker implantation(n,%) | 8(13.79) | 4(11.43) | | ≥0.999 | |
| Stent eccentric index(%) | 16.97 ± 9.28 | 10.92 ± 9.87 | | 0.025 | |
| Echocardiographic parameters before discharge |  |  | |  | |
| LVEF(%) | 58.23 ± 9.27 | 60.97 ± 7.43 | | 0.155 | |
| Peak velocity(m/s) | 2.25 ± 0.49 | 2.32 ± 0.69 | | 0.610 | |
| Mean gradient(mmHg) | 11.95 ± 4.66 | 12.69 ± 6.30 | | 0.520 | |
| EOA(mm²) | 2.14±0.53 | 2.27±0.60 | | 0.319 | |

Abbreviations were shown as above.
